# TP53INP1 inhibits hypoxia‐induced vasculogenic mimicry formation via the ROS/snail signalling axis in breast cancer

**DOI:** 10.1111/jcmm.13625

**Published:** 2018-04-14

**Authors:** Yi Wang, Huizhi Sun, Danfang Zhang, Dan Fan, Yanhui Zhang, Xueyi Dong, Shiqi Liu, Zhao Yang, Chunsheng Ni, Yanlei Li, Fang Liu, Xiulan Zhao

**Affiliations:** ^1^ Department of Pathology Tianjin Medical University Tianjin China; ^2^ Department of Pathology General Hospital of Tianjin Medical University Tianjin China; ^3^ Department of Pathology Cancer Hospital of Tianjin Medical University Tianjin China

**Keywords:** breast cancer, epithelial‐mesenchymal transition, hypoxia, TP53INP1, vasculogenic mimicry

## Abstract

Tumour protein p53‐inducible nuclear protein 1 (TP53INP1) is a tumour suppressor associated with malignant tumour metastasis. Vasculogenic mimicry (VM) is a new tumour vascular supply pattern that significantly influences tumour metastasis and contributes to a poor prognosis. However, the molecular mechanism of the relationship between TP53INP1 and breast cancer VM formation is unknown. Here, we explored the underlying mechanism by which TP53INP1 regulates VM formation in vitro and in vivo. High TP53INP1 expression was not only negatively correlated with a poor prognosis but also had a negative relationship with VE‐cadherin, HIF‐1α and Snail expression. TP53INP1 overexpression inhibited breast cancer invasion, migration, epithelial‐mesenchymal transition (EMT) and VM formation; conversely, TP53INP1 down‐regulation promoted these processes in vitro by functional experiments and Western blot analysis. We established a hypoxia model induced by CoCl_2_ and assessed the effects of TP53INP1 on hypoxia‐induced EMT and VM formation. In addition, we confirmed that a reactive oxygen species (ROS)‐mediated signalling pathway participated in TP53INP1‐mediated VM formation. Together, our results show that TP53INP1 inhibits hypoxia‐induced EMT and VM formation via the ROS/GSK‐3β/Snail pathway in breast cancer, which offers new insights into breast cancer clinical therapy.

## 1 INTRODUCTION

Breast cancer is the second most common cause of cancer‐related death in women worldwide.^1^ Most advanced breast cancer patients die primarily as a result of distant metastasis. The rapid growth of tumour cells leads to hypoxia in the tumour microenvironment, and hypoxia can further promote breast cancer development and metastasis.^2^, ^3^ Molecular mechanisms to inhibit breast cancer metastasis in a hypoxic environment should thus be elucidated; the inhibition of hypoxia‐induced related pathways is predicted to be a necessary anticancer treatment target in breast cancer.

Vasculogenic mimicry (VM), a newly discovered blood supply model, is a vessel structure made of cancer cells and lacking endothelial cells.^4^ A hypoxic microenvironment not only leads to VM formation but also accelerates tumour metastasis, increasing the risk of resistance to chemotherapy in breast cancer.^5^, ^6^ The expression of hypoxia‐inducible factor‐1α (HIF‐1α) has been reported to be associated with VM in many cancers types, including liver cancer,^7^ ovarian cancer,^8^ colorectal cancer^9^ and melanoma.^10^ These observations suggest a new idea for inhibiting VM formation in hypoxia.

Epithelial‐mesenchymal transition (EMT) has also been confirmed to be associated with breast cancer cell invasion and is a key step for VM formation.^11^ Research has shown that HIF‐1α can influence EMT‐related gene expression, resulting in a cell phenotype change.^12^ Furthermore, reactive oxygen species (ROS) accumulated in the mitochondria could activate the EMT transcription factor Snail to promote cancer progression and participate in the regulation of HIF‐1α transcriptional activity.^13^ We speculated that hypoxia‐inducible EMT may be indispensable in VM formation in breast cancer.

Tumour protein 53‐induced nuclear protein 1 (TP53INP1), also called stress‐induced protein (SIP), is a tumour suppressor gene located on chromosome 8q22.^14^, ^15^ TP53INP1 is a major regulator of p53 in response to oxidative stress, including hypoxia.^16^ TP53INP1‐deficient cells generate more intracellular ROS.^17^ TP53INP1 participates in cancer progression in p53‐independent and p53‐dependent processes.^14^, ^18^ As a target gene of p53, TP53INP1 regulates p73 activity by binding to p53‐responsive elements.^19^, ^20^, ^21^ Moreover, it has been reported that TP53INP1 can modulate p21,^22^ HIPK2,^23^ SPARC^24^ and TGF‐β,^25^ suggesting that TP53INP1 plays a critical role in cancer progression. TP53INP1 also negatively regulates the metastasis of malignant tumours including cancers of gliocytes,^26^ breast,^27^ the stomach,^28^ liver^29^ and pancreas.^30^ The loss or low expression of TP53INP1 is positively associated with breast cancer progression.^27^

In our study, we investigated the role of TP53INP1 in breast cancer cells (BCC) EMT and VM formation under hypoxic conditions and preliminarily explored the ROS/Snail signalling pathway in TP53INP1‐mediated VM formation. These findings indicate a new functional role for TP53INP1 in breast cancer and provide insight for BCC anti‐angiogenic drugs.

## 2 MATERIALS AND METHODS

### 2.1 Patient samples

One hundred random breast cancer specimens were obtained from the General Hospital of Tianjin Medical University (Tianjin, China). These specimens were collected from patients between 1997 and 2005. A diagnosis of breast cancer in these samples was verified by two or more pathologists. Detailed pathological and clinical data were collected for all samples. The use of these tissue samples was approved by the Ethics Committee of Tianjin Medical University.

### 2.2 Immunohistochemistry and CD31/PAS double staining

Immunohistochemistry was performed on paraffin‐embedded sections of 100 breast cancer tissues. Tissues were dewaxed and rehydrated using graded concentrations of alcohol. Tissue sections were then pre‐treated by microwave retrieval, blocked with goat serum and incubated with primary and secondary antibodies. DAB staining was performed for appropriate durations, and all sections were counterstained with haematoxylin. After CD31 staining was performed, the tissue was incubated with 0.5% periodic acid for 15 minutes and Schiff's stain for 15 minutes for double staining. PBS was used in place of the primary antibodies for all negative controls. The staining intensity was scored based on four classes: 0 (negative), 1 (weak), 2 (medium) and 3 (high). The percentage of stained cells in the whole field was scored as follows: 0 (negative), 1 (≤25%), 2 (25%‐50%) and 3 (>50%). The final score, determined by adding the intensity and percentage above two scores together, was grouped as follows: ≤3 (negative) and >3 (positive).

### 2.3 Cell culture

The human breast cancer cell lines MDA‐MB‐231 and MCF‐7 were purchased from ATCC in 2012 and were certified in 2014. The two cell lines were cultured in DMEM medium supplemented with 10% foetal bovine serum (FBS; Pro) and 1% antibiotics (penicillin and streptomycin) in a humidified atmosphere. The cultures were treated with 150 μmol/L cobalt chloride (CoCl_2_) and incubated for 48 hours in 6‐well plates to mimic a hypoxic environment. N‐acetyl‐cysteine (NAC; 20 mmol/L) (Sigma‐Aldrich, MO, USA), an oxidant scavenger, was added to transfected cells.

### 2.4 Expression plasmids and gene silencing

A stable transfected MDA‐MB‐231 cell line was obtained using lentiviral expression plasmids with TP53INP1 cDNA (catalogue no. EX‐T1715‐Lv201) or a negative control (catalogue no. EX‐NEG‐Lv201). MCF‐7 cells were transfected with TP53INP1 shRNA (HSH022738‐LvRU6MP) or shRNA control (CSHCTR001‐LVRU6MP) purchased from GeneCopoeia, Inc. These cell lines were transfected using the Lenti‐Pac™ HIV Expression Kit (Genecopia Co.) according to the manufacturer's protocol. After transfection of the 293T cells with the lentiviral expression plasmids, the virus‐containing medium was used to infect the experimental cells and created stable cell lines via puromycin selection.

### 2.5 Western blotting analysis

Protein was extracted using SDS lysis buffer and transferred to PVDF membranes. After the membranes were blocked with 5% skim milk for 1 hour, they were incubated with primary antibodies overnight at 4°C, which was followed by incubation with secondary antibodies for 2 hours. Bands were visualized using a C‐Digit Blot Scanner (Gene Company) and analysed with ImageJ software. β‐actin (sc1616‐R, 1:1000; Santa Cruz) was used as a protein loading control. The antibodies are listed in Table S1.

### 2.6 Immunofluorescence staining

Cells in the different groups were seeded onto glass slides. When the cells were grown to 50%‐60% confluency, they were washed with PBS and fixed with cold methanol at −20°C. The cells were permeabilized with 0.1% Triton X‐100 in PBS for 20 minutes and blocked with 5% FBS in PBS at room temperature for 30 minutes. The cells were incubated with primary antibodies for 1 hour at 37°C and overnight at 4°C. The cells were then incubated with secondary antibodies for 1 hour and washed with PBS. Nuclear staining with DAPI (Sigma) was then performed. Slides were viewed under a fluorescence microscope (Nikon, Japan).

### 2.7 Wound‐healing assay

Cells were seeded in 6‐well plates. When cells reached confluency, a wound was created using a 100‐μL sterile pipette tip and photographed (0 hour). The rate of gap closure was measured at different time‐points. Each experiment was performed three times.

### 2.8 Cell invasion assay and cell migration assays

Migration assays were performed with breast cancer cells (1 × 10^5^) that were added to the upper chamber with serum‐free medium, and DMEM with 10% FBS was added to the bottom chamber in 24‐well plates. After the cells were incubated for 24 hours, they were fixed with methanol and stained with crystal violet for 20 minutes. Invasion assays were performed as with the migration assays except that the transwells chambers were coated with Matrigel before the cells were seeded in the upper chamber. These cells were counted using an inverted light microscope (Nikon). Each experiment was performed three times.

### 2.9 Three‐dimensional (3‐D) cultures

For 3D culture, 96‐well plates were coated with Matrigel (BD, USA) on ice, and the gel was incubated for 1 hour at 37°C. A breast cancer cell suspension (10^5 ^cells/well) with or without CoCl_2_ (150 μmol/L) was added into the plates when the gel solidified, and the plates were placed into a 37°C 5% CO_2_ incubator for 24 hours. Channel‐like structure was selected in random fields and filmed under phase contrast microscopy (100×); each group experiment was performed at least three times.

### 2.10 ROS measurement

Breast cancer cells were seeded at a density of 2 × 10^5^ cells/well in a 6‐well plate and were treated with drugs for 48 hours at 37°C in 5% CO_2_. DCFH‐DA (20 μmol/L; Sigma D6883) was diluted in culture medium and incubated for 30 minutes in a humidified atmosphere. Cells were washed three times with PBS, suspended in flow tubes, and then analysed with a FACS Accuri C6 (BD Biosciences America). ROS generation was measured relative to the control, and 1 × 10^5^ cell events were analysed for each group.

### 2.11 Reverse transcriptase polymerase chain reaction(RT‐PCR)

Total RNA from the adhesive breast cancer cells in a 6‐well plate was extracted with TRIzol Reagent according to manufacturer's protocol (TIANGEN, China) and was reverse transcribed into cDNA using the RT‐PCR Kit (TIANGEN, China). RT‐PCR was performed with a gradient thermal cycler (Gene Company Limited, HK). The PCR conditions were 95°C for 30 seconds followed by 40 cycles of 95°C for 30 seconds, 60°C for 1 minute and 72°C for 30 seconds following. PCR products were electrophoretically on a Sepharose gel, visualized with a gel document system (Syngene, UK) and analysed by ImageJ software. The sequences of primer are listed in Table S2.

### 2.12 Xenografts and treatments

Tientsin Albino 2 (TA2) mice (weighing 18‐22 g, 4‐6 weeks) from the Animal Center of Tianjin Medical University develop spontaneous breast cancer, and they were used to establish an in vivo model. We selected five different tumours, and two of these were approximately equivalent to the MDA‐MB‐231 and MCF‐7 cell lines. The cancer cells (5 × 10^6^ cells) were injected subcutaneously into the rat groin region of the TA2 mice. When the tumours reached 0.4 cm^3^, plasmids along with the DNA transfection reagent (Entranster‐in vivo, Engreen) were injected into the tumour every 3 days. We measured the change in tumour volume (width^2^ × length/2). These TA2 mice were simultaneously killed when the volume of tumours showed significant difference. Subsequently, we conducted immunohistochemistry analysis and endomucin/PAS double staining. All experiments were authorized by the Tianjin Medical University Institutional Animal Care and Use Committee (IACUC).

### 2.13 Statistical analysis

All experiments were repeated independently at least three times. Values are presented as the means ± SD. Analyses were performed with SPSS 22.0 (SPSS Inc, Chicago, IL, USA) statistical analysis software. Student's *t* test was used to determine differences between two groups. Measurement data among three or more groups were compared by ANOVA. The survival of each group was evaluated by Kaplan‐Meier analysis. Statistical significance was defined as *P *<* *.05.

## 3 RESULTS

### 3.1 Clinical significance of TP53INP1 in human breast cancer patients

**Figure 1:**
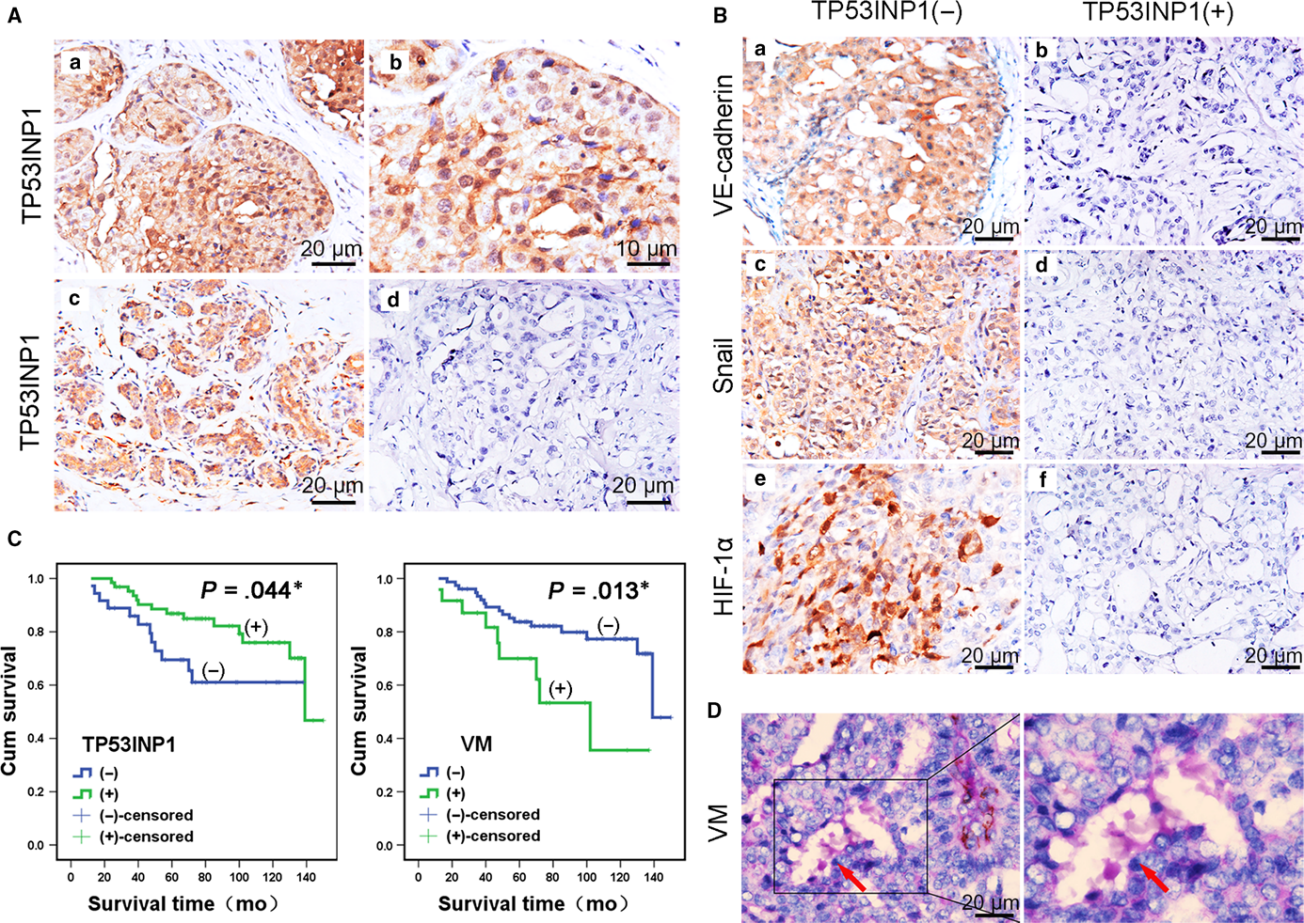
TP53INP1 is correlated with VM in human breast cancer tissues A, Immunohistochemistry showed that TP53INP1 was mainly located in the cytoplasm and nucleus in breast cancer specimens and adjacent tissues. Positive expression of TP53INP1 (a, b, 200× and 400×); negative expression of TP53INP1 (d, 200×) in cancer specimens; positive expression of TP53INP1 in adjacent tissues (c, 200×). B, Positive expression and negative expression of VE‐cadherin (a, b), Snail (c, d) and HIF‐1α (e, f) in breast cancer tissue specimens by IHC (200×). C, Kaplan‐Meier survival curves of patients with breast cancer showed that TP53INP1 expression was negatively correlated with poor prognosis (*P *=* *.044), and positive VM was correlated with poor prognosis (*P *=* *.013). *P < .05 D, VM phenomenon in TP53INP1‐negative breast cancer tissue (red arrow, CD31‐negative and PAS‐positive; 400× and 720×); all *P *<* *.05

To investigate TP53INP1 expression in human breast cancer tissues, we analysed 100 breast cancer samples by immunohistochemistry. TP53INP1 was mainly located in the cytoplasm and nucleus. High TP53INP1 expression was found in 64 of 100 breast cancer sample tissues (64%), and low expression was identified in 36 of 100 (36%) samples (Table 1, Figure 1A and S1). We found that 73 of 100 breast cancer samples had accompanying pericarcinous tissues; of these, the high expression of TP53INP1 was 49.3% (36/73) in breast cancer tissues and 65.8% (48/73) in matched adjacent tissues. Thus, TP53INP1 expression was lower in breast cancer tissues than in pericarcinous tissues (*P *=* *.045;Table 2). Studying further the correlation between TP53INP1 expression and clinical prognosis, we found that TNM stage exhibited a significant difference between the high TP53INP1 expression group and the low expression group; low TP53INP1 expression was found in 13 of 21 TNM stage III/IV samples (*P *=* *.005). Similarly, low TP53INP1 expression was positively correlated with lymphatic metastasis (*P *=* *.015) and triple‐negative status (*P *=* *.012; Table 1). Meanwhile, we observed that TP53INP1‐negative tumour cells had relationship with VM formation. Only 10 samples with CD31‐negative staining and PAS‐positive staining showed VM tubes, but 54 samples in the TP53INP1 overexpression group showed no VM tubes (*P *=* *.009; Table 1), indicating that the expression of TP53INP1 was negatively correlated with VM. In addition, Kaplan‐Meier survival analysis showed that low TP53INP1 expression correlated with poor overall survival (*P = *.044), and high VM expression was correlated with poor overall survival (*P = *.013; Figure 1C). The group with a combination of TP53INP1 low expression and VM positivity had a worse prognosis than the other groups (*P *=* *.001; Figure S2A). These results suggest that TP53INP1 expression may be associated with VM in breast cancer.

**Table 1:** Relationship between TP53INP1 expression and clinicopathologic features, VM formation in breast cancer

Variables	TP53INP1		χ²	*P*
	−(%)	+(%)		
Age				
<50	23	38	0.197	.657
≥50	13	26		
Tumour size				
D < 2	6	11	0.355	.838
2 ≤ D ≤ 5	28	51		
D > 5	2	2		
Grade				
I/II	24	48	0.794	.373
III	12	16		
TNM stage				
I/II	23	56	7.742	.005^a^
III/IV	13	8		
Lymphatic metastasis				
No	14	41	5.899	.015^a^
Yes	22	23		
Triple‐negative				
No	24	56	6.250	.012^a^
Yes	12	8		
VM				
No	22	54	6.836	.009^a^
Yes	14	10		

a Statistically significant *P *<* *.05.

VM, vasculogenic mimicry.

**Table 2:** TP53INP1 expression in breast cancer tissues and pericarcinous tissues

	Cancer tissue N = 73	Pericarcinous tissue N = 73	χ²	*P*
TP53INP1				
Negative	37	25	4.037	.045^a^
Positive	36	48		

a Statistically significant *P *<* *.05.

### 3.2 TP53INP1 is associated with VE‐cadherin, Snail and HIF‐1α expression in human breast cancer tissues

Statistical analysis showed that VE‐cadherin was highly expressed in 26 of 36 (72.2%) TP53INP1‐negative breast cancer tissues (*P = *.044; Figure 1Ba), and there was a negative correlation between TP53INP1 and VE‐cadherin (Pearson analysis *r* = −.0227, *P* = .023;Figure S2B). Similarly, 29 and 24 of 36 samples (80.6%, 66.7%) with low TP53INP1 expression overexpressed Snail (*P = *.001) and HIF‐1α (*P = *.012;Figure 1Bc‐f), respectively. The combination of the low TP53INP1 expression and HIF‐1α overexpression group had a worse prognosis than did the other groups (*P = *.022; Figure S2C). In addition, we also observed a correlation of TP53INP1 expression with the expression of HIF1‐α in breast cancer by Pearson analysis (*r* = −.0293, *P *=* *.003; Figure S2D). These data showed that TP53INP1 was negatively correlated with VE‐cadherin, Snail and HIF‐1α expression (Table 3). Together, these results indicated that TP53INP1 may be correlated with hypoxia‐induced VM.

**Table 3:** Difference of VE‐cadherin, Snail and HIF‐1α expression in TP53INP1‐positive and TP53INP1‐negative groups

Variant	TP53INP1		χ²	*P*
	−(%)	+(%)		
VE‐cadherin				
Negative	10	31	4.065	.044^a^
Positive	26	33		
Snail				
Negative	7	34	9.032	.001^a^
Positive	29	30		
HIF1‐α				
Negative	12	38	6.250	.012^a^
Positive	24	26		

a Statistically significant *P *<* *.05.

### 3.3 TP53INP1 inhibits breast cancer cell VM formation in vitro

The expression of TP53INP1 was lowest in MDA‐MB‐231 cells compared with three other BCC lines (T‐47D, HS‐578T and MCF‐7). MDA‐MB‐231 cells are a triple‐negative breast cancer line that can form VM channels. In contrast, MCF‐7 cells show high TP53INP1 levels by Western blot (Figure S3A). Using the lentiviral constructs and plasmid infection, the overexpression of TP53INP1 in MDA‐MB‐231 cells and low TP53INP1 expression in transfected MCF‐7 cells were confirmed. Both cell lines were transfected with non‐target plasmids to serve as controls (Figure S3B).

**Figure 2:**
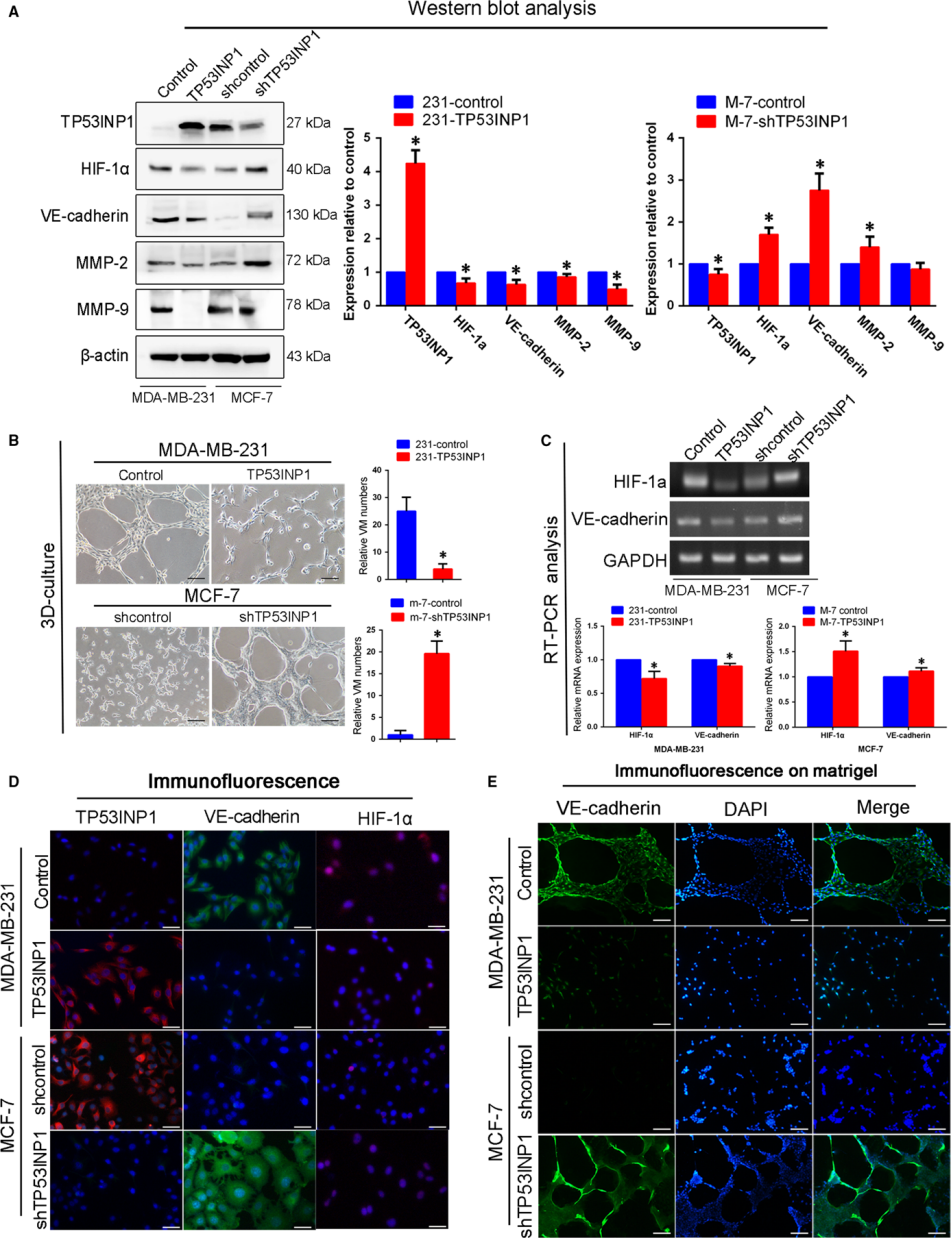
TP53INP1 inhibits breast cancer cells VM formation in vitro A, The expression levels of TP53INP1, HIF‐1α, VE‐cadherin, MMP2 and MMP9 were evaluated by Western blotting samples from TP53INP1‐overexpressing and shTP53INP1‐expressing breast cancer cells. B, VM channel formation in TP53INP1‐overexpressing MDA‐MB‐231 and shTP53INP1‐expressing MCF‐7 cells (100×). C, D, The expression levels of TP53INP1, VE‐cadherin and HIF‐1α were verified by RT‐PCR and immunofluorescence staining. E, Immunofluorescence staining was performed after a 3D culture assay to observe the expression of VE‐cadherin. TP53INP1‐overexpressing MDA‐MB‐231 cells did not form VM channels, and VE‐cadherin expression decreased compared with the control group. Accordingly, shTP53INP1 cell lines formed VM channels, and VE‐cadherin expression increased compared with the control (100×). These results are expressed as the means ± SD of at least three separate experiments. *P <* *.05

To determine whether the expression of TP53INP1 was associated with VM in vitro, VE‐cadherin expression was monitored; it was lower in TP53INP1‐overexpressing MDA‐MB‐231 cells than in control cells. The expression levels of HIF‐1α, MMP2 and MMP9 were also down‐regulated. VE‐cadherin, MMP2 and HIF‐1α protein levels increased in TP53INP1‐silenced MCF‐7 cells, but there was no change in MMP9 expression (Figure 2A). We used three‐dimensional cell cultures to demonstrate the ability of the cells to form channels. The ability of TP53INP1‐overexpressing MDA‐MB‐231 cells to form a channel‐like structure was weaker than that of control cells. In contrast, TP53INP1 down‐regulation in MCF‐7 cells had the opposite result (Figure 2B). RT‐PCR analysis and immunofluorescence results both confirmed these results (Figure 2C,D). To further demonstrate that TP53INP1 could control the formation of VM, both MDA‐MB‐231 control cells and TP53INP1‐silenced MCF‐7 cells exhibited the ability to form VM‐like networks on Matrigel and showed high expression of VE‐cadherin, which is consistent with the above results (Figure 2E). These data demonstrated that TP53INP1 inhibited VM formation in breast cancer cells.

### 3.4 TP53INP1 attenuates the migration and invasion capacity, and TP53INP1 down‐regulation is associated with EMT

**Figure 3:**
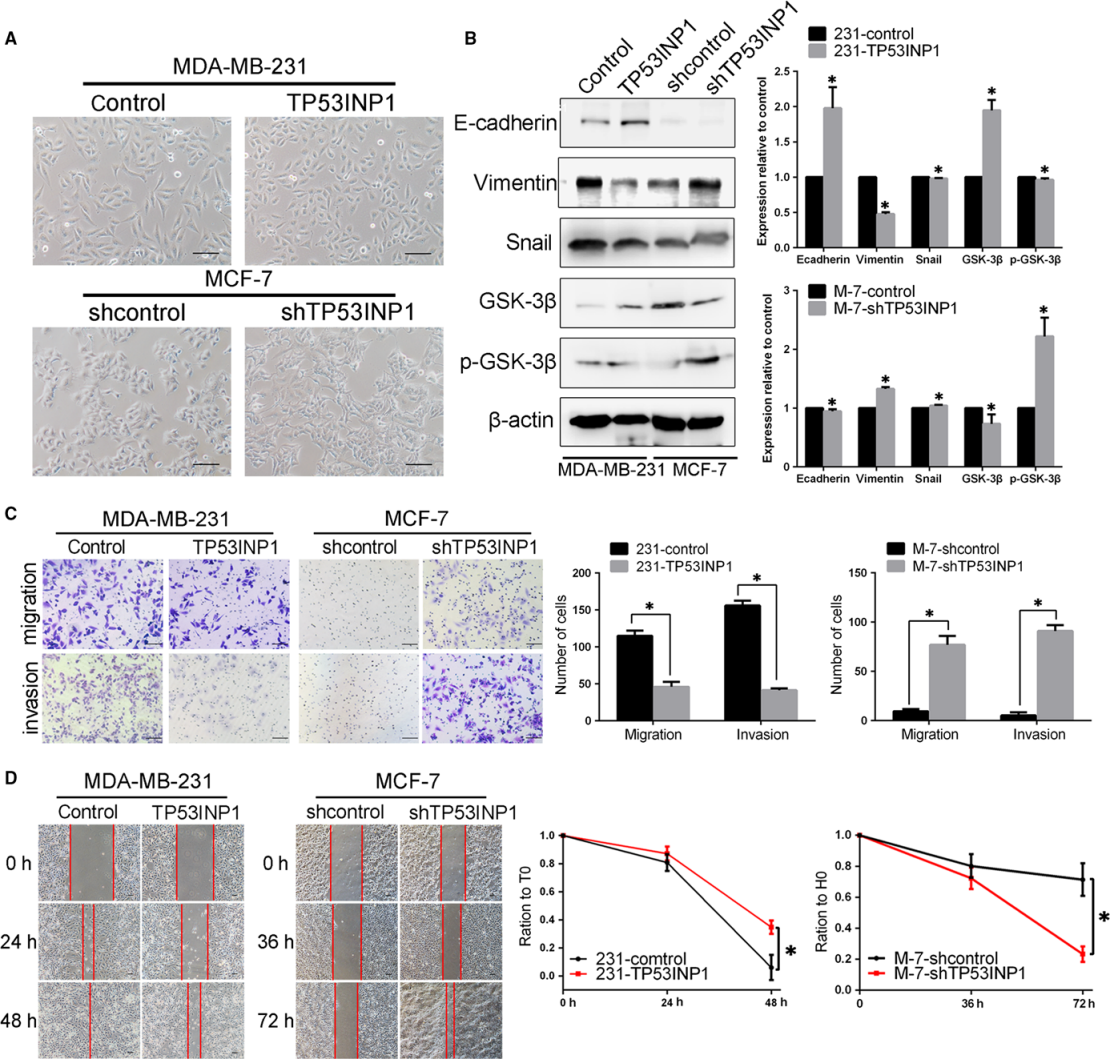
TP53INP1 attenuates cancer cell migration and invasion, and TP53INP1 down‐regulation is associated with EMT A, The cell morphology transformation induced by silencing TP53INP1 in MCF‐7 cells and by overexpressing TP53INP1 in MDA‐MB‐231 cells (100×). B, The expression levels of EMT‐related markers were evaluated by Western blotting in TP53INP1‐transfected breast cancer cells. C, D, The migration and invasion assay (100×) and wound‐healing assay (40×) were conducted in these TP53INP1‐transfected cells. These data are presented as the means ± SD. Each experiment was performed in three individual replicates. *P <* *.05

We sought to determine whether TP53INP1 overexpression would be sufficient to inhibit EMT in breast cancer cells. We first observed that silencing TP53INP1 in MCF‐7 cells resulted in a spindle‐like, fibroblastic morphology, whereas there was no obvious morphology change in TP53INP1‐overexpressing MDA‐MB‐231 cells (Figure 3A). Western blot results showed that the expression of E‐cadherin and GSK‐3β was up‐regulated, whereas vimentin, Snail and phospho‐GSK‐3β levels were decreased by the overexpression of TP53INP1 (Figure 3B). The immunofluorescence results were consistent with the Western blotting results (Figure S4). Next, we have been suggested that TP53INP1 could inhibit BCC migration in vitro. The results showed that compared with the control, overexpressing TP53INP1 resulted in fewer migrated cells. However, TP53INP1‐silenced MCF‐7 cells migrated faster than controls (Figure 3C). Wound‐healing assays also confirmed these results; TP53INP1 decreased the healing rate of the wound (Figure 3D). In general, these results indicated that TP53INP1 not only attenuates breast cancer cell migration and invasion but also inhibits EMT, which suggests that TP53INP1 is related to VM formation.

### 3.5 Inhibiting TP53INP1 promotes hypoxia‐induced EMT and VM formation in breast cancer cells, and its expression decreases hypoxia‐induced migration and invasion

**Figure 4:**
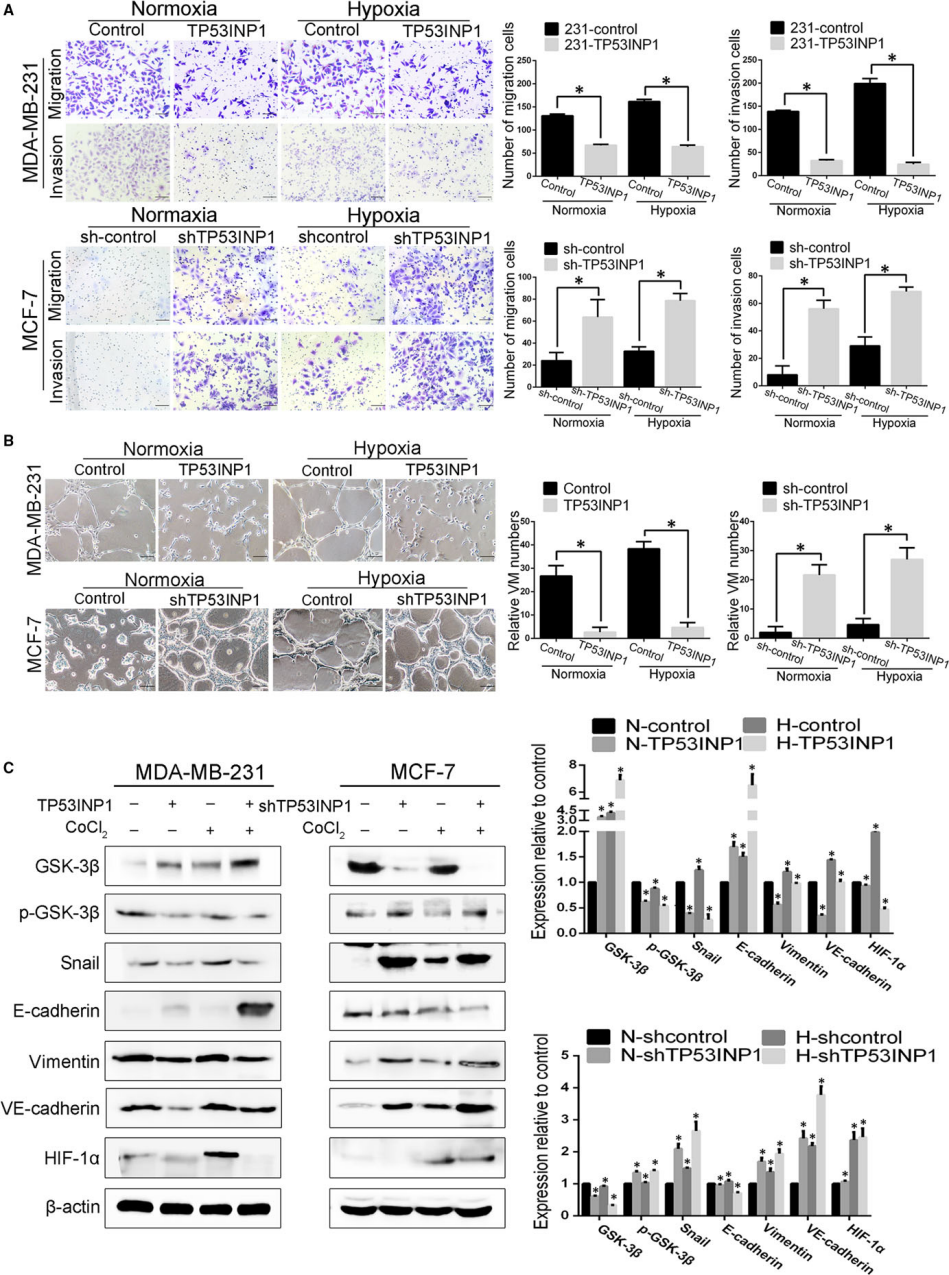
Inhibiting TP53INP1 promotes hypoxia‐induced VM formation and EMT in breast cancer cells A, The migration and invasion assays were performed in TP53INP1‐overexpressing MDA‐MB‐231 cells and TP53INP1‐silenced cells in the presence of CoCl_2_ treatment (150 μmol/L 48 h, 100×). B, TP53INP1 inhibited VM formation with CoCl_2_ treatment on Matrigel (100×). C, The protein level of EMT‐ and VM‐related markers was tested by Western blotting samples from TP53INP1‐overexpressing MDA‐MB‐231 cells and TP53INP1‐silenced MCF‐7 cells with CoCl_2_ treatment. Data are presented as the means ± SD. Each condition was carried out with three individual replicates. *P < 0.05

To further evaluate the influence of TP53INP1 on hypoxia‐induced VM formation in breast cancer, we again assessed migration and invasion. TP53INP1‐overexpressing cells showed decreased migration and invasion, whereas shTP53INP1 MCF‐7 cells migrated more rapidly than control cells in a hypoxic model (Figure 4A). Next, we found that TP53INP1‐overexpressing breast cancer cells formed very few tube‐like structures in the hypoxic environment, whereas the control group formed more VM channels. However, TP53INP1 knock‐down facilitated the formation of VM channels compared with the control with CoCl_2_ treatment (Figure 4B). These results were consistent with those in normoxia. We also revalidated that breast cancer cells exposed to hypoxia had increased ability to form channels and increased invasive capacity. As shown by the results of Western blotting assays, we found the up‐regulation of E‐cadherin and GSK‐3β, and the down‐regulation of phospho‐GSK‐3β, vimentin and Snail in CoCl_2_‐treated TP53INP1‐overexpressing cells compared to control cells. The expression of VE‐cadherin and HIF‐1α was also reduced. However, MCF7‐shTP53INP1 cells had opposite results, which promoted EMT‐related protein expression. Meanwhile, we also verified that EMT and VM‐related protein levels in breast cancer cells increased in a hypoxic environment compared with those in a normoxic environment (Figure 4C). To further verify that HIF‐1α inhibits TP53INP1 expression, we performed a rescue experiment in which HIF‐1α was down‐regulated in TP53INP1‐overexpressing cells and TP53INP1‐silenced cells. Cell with shHIF‐1α treatment showed increased TP53INP1 expression compared to the control group. The protein levels of VE‐cadherin and MMP2 decreased in TP53INP1‐overexpressing cells independent of shHIF‐1α treatment. Cells with TP53INP1 knock‐down that were treated with shHIF‐1α or CoCl_2_ exhibited increased VE‐cadherin and MMP2 protein levels (Figure S5). These results suggested that TP53INP1 inhibits hypoxia‐induced downstream effectors that promote EMT and VM formation in a hypoxic microenvironment, and TP53INP1 may be mediated by HIF‐1α in breast cancer cells.

### 3.6 A ROS‐mediated signalling pathway participates in the inhibition by TP53INP1 of hypoxia‐induced VM formation and EMT in vitro

**Figure 5:**
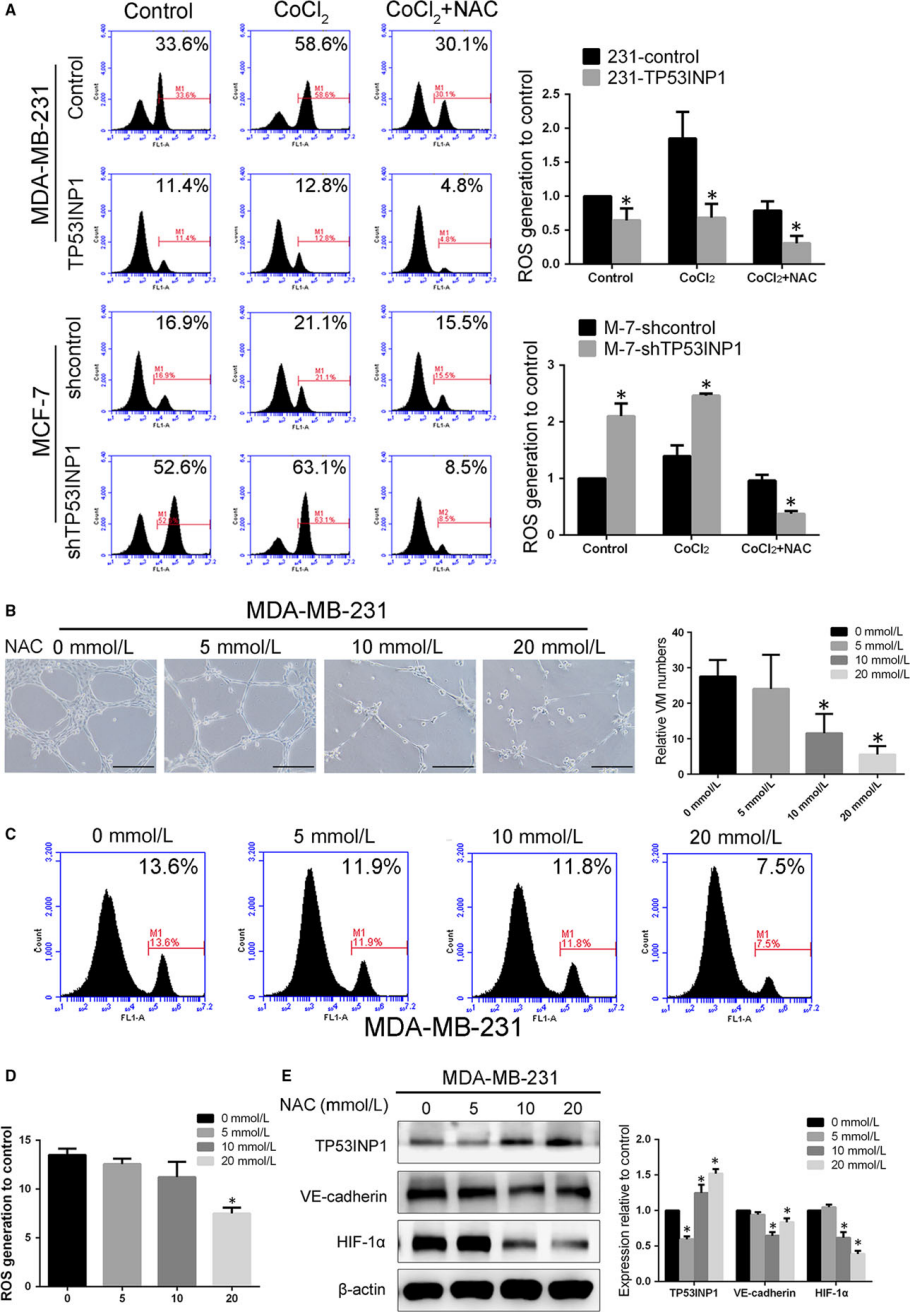
A ROS‐mediated signalling pathway participates in TP53INP1‐mediated VM formation and EMT in vitro A, ROS level measurement in the different treatment groups by flow cytometry. *P <* *.05 B, NAC (20 mmol/L) inhibited VM channel formation in MDA‐MB‐231 cells in 3D culture. (100×). C, D, ROS generation was measured in MDA‐MB‐231 cells treated with various concentrations of NAC for 48 h and analysed by flow cytometry. High‐level ROS production was seen in 7.5% of cells with 20 mmol/L NAC treatment, which was lower than that in the other groups. E, The protein levels of TP53INP1, VE‐cadherin and HIF‐1α in MDA‐MB‐231 cells after treatment with various concentrations of NAC for 48 h. Treatment with 20 mmol/L NAC decreased the levels of VE‐cadherin and HIF‐1α. Data are presented as the means ± SD. Each condition was repeated three times. *P <* *.05

To confirm that TP53INP1 inhibits EMT and VM formation by mediating the activation of ROS signalling in response to hypoxia, we first evaluated the ROS level by flow cytometry analysis. We found that the treatment of MDA‐MB‐231 and MCF‐7 cells with 150 μmol/L CoCl_2_ increased ROS levels compared with the control, but NAC (an oxidant scavenger) attenuated the CoCl_2_‐induced increase in ROS. And the ROS level in TP53INP1‐overexpressing cells was not affected by treatment with CoCl_2_ or NAC. Interestingly, we found that the ROS level was decreased compared to the control group with or without CoCl_2_ treatment after TP53INP1 up‐regulation. Consistently, the levels of ROS accumulation were higher in the TP53INP1‐silenced MCF‐7 cells under hypoxia or normoxia than in the control groups (Figure 5A). Then, we analysed VM channel formation and ROS generation with the different NAC concentrations in MDA‐MB‐231 cells; 20 mmol/L NAC completely inhibited the VM channel formation and decreased ROS levels (Figure 5B‐D). Additionally, the protein levels of VE‐cadherin and HIF‐1α were significantly decreased, and TP53INP1 was increased after 20 mmol/L NAC inhibition (Figure 5E). These results indicated the role of ROS in BBC VM formation. To further validate the role of the ROS signalling pathway in TP53INP1‐regulated cell lines, we analysed the effect of TP53INP1 expression with NAC and found that EMT‐ and VM‐related protein expression levels were not changed in TP53INP1‐overexpressing MDA‐MB‐231 cells regardless of NAC or CoCl_2_ treatment. shTP53INP1 MCF‐7 cells were treated with NAC and CoCl_2,_ and NAC was confirmed to inhibit the increase in the CoCl_2_‐induced EMT and VM‐related protein levels (Figure S6). These data indicated that the role of ROS could be affected by TP53INP1, which promotes EMT and VM formation in breast cancer cells.

### 3.7 TP53INP1 inhibits breast cancer growth, invasion and VM formation in the TA2 mouse model

**Figure 6:**
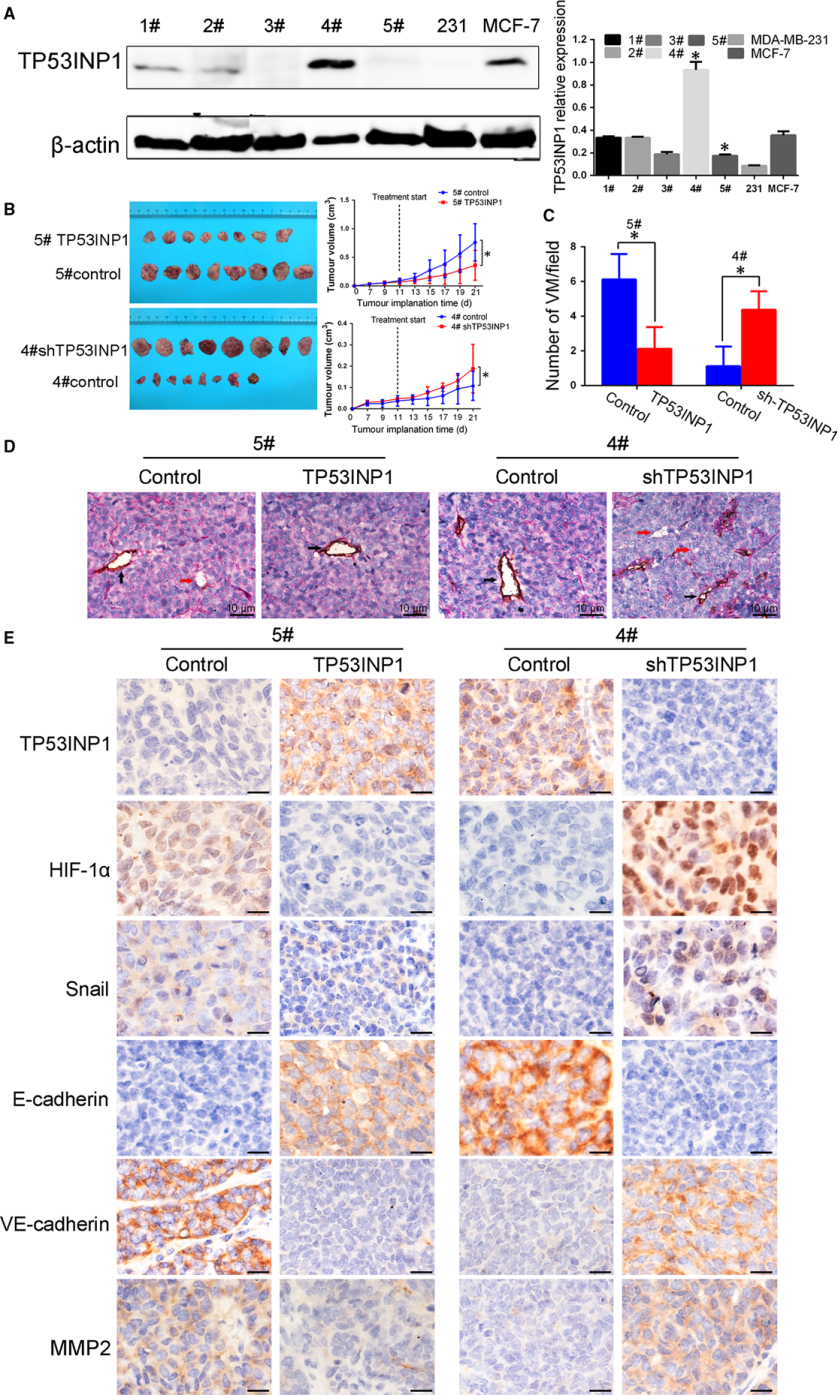
TP53INP1 inhibits breast cancer growth, invasion and VM formation in the TA2 mouse model A, The selection of two spontaneous TA2 breast cancers out of five; the TP53INP1 expression was consistent with the MDA‐MB‐231 and MCF‐7 cell lines based on Western blots. B, Tumour growth curves showed TP53INP1 overexpression inhibits tumour growth. C, D, VM formation in TA2 tumours from indicated groups revealed by an endomucin/PAS double‐staining method (400×). E, The expression levels of TP53INP1, HIF‐1α, Snail, E‐cadherin, VE‐cadherin and MMP2 were evaluated in these indicated groups by IHC staining (400×). *P <* *.05

To further clarify the relationship between TP53INP1 and VM formation in breast cancer, we explored the effect of TP53INP1 plasmid infection in vivo. First, we selected two of five spontaneous TA2 breast cancers; the TP53INP1 expression in these lines showed obvious differences by Western blotting. The group that was consistent with the MDA‐MB‐231 cell line was considered as the high‐metastasis group and was infected with the TP53INP1‐overexpressing plasmid; conversely, the other group was considered the low‐metastasis group and was infected with the shTP53INP1 plasmid (Figure 6A). Ten days after injecting plasmid, the tumour sizes were obviously smaller in the TP53INP1‐overexpressing group than in the control group. In contrast, after injection with the shTP53INP1 plasmid, the tumour growth in TA2 mice was greater than that in the control mice (*P *<* *.05, Figure 6B). In addition, based on the results of endomucin/PAS double‐staining, we confirmed that VM channel formation decreased in the TP53INP1‐overexpressing mice. However, more VM channel formation was observed in the TP53INP1‐silenced group than in the control group (Figure 6C,D). Finally, immunohistochemistry results indicated that the expression levels of Snail, VE‐cadherin, MMP2 and HIF‐1α were reduced in tumours with TP53INP1 overexpression, and the expression of E‐cadherin was increased. Tumours in the TP53INP1‐silenced group expressed more Snail, VE‐cadherin, MMP2 and HIF‐1α protein than the control group tumours (Figure 6E). Together with the in vitro experiments, we conclude that TP53INP1 inhibits breast cancer EMT and VM formation in vivo.

## 4 DISCUSSION

TP53INP1 is a p53‐driven oxidative stress response protein that is induced in many different stress situations, such as oxidative stress, radiation and inflammation.^31^, ^32^ TP53INP1 was shown to be associated with homeodomain‐interacting protein kinase‐2 (HIPK2) to mediate the antioxidant activity of p53.^16^, ^23^ The antioxidant function of TP53INP1 stems from the control of mitochondrial ROS production; the absence of TP53INP1 enhances ROS production.^17^, ^31^ It has been reported that TP53INP1 can induce the transcription of both p53 and p73 in the nucleus. TP53INP1 is both a target gene and a regulator of p53. A p53‐deficient cell line showed decreased TP53INP1 expression due to activated p73 transcriptional activity.^14^, ^18^ The transcription factor E2F1 increases TP53INP1 expression during cell cycle progression.^33^ TP53INP1 exerts antitumour effects through a p53‐independent pathway in breast cancer. Liu et al found that TP53INP1 decreases liver cancer cell migration and invasion by inhibiting EMT.^25^ The down‐regulation of TP53INP1 promotes the metastasis of hepatocellular carcinoma through the p73‐mediated ERK signalling pathway.^34^ TP53INP1 can decrease cell migration by regulating SPARC expression in pancreatic cancer.^24^ TP53INP1 expression is decreased in metastatic brain tumours from the lung.^35^ In this study, our data confirmed that the role of TP53INP1 was negatively associated with VM in breast cancer.

Triple‐negative breast cancer (TNBC) is a breast cancer subtype which is aggressive, metastasizes to distant organs and has a poor prognosis. Our previous study showed greater VM formation in TNBC patients than in non‐TNBC patients, which supports the conclusion that VM formation is positively associated with TNBC.^36^, ^37^ Our data demonstrated that there is a relationship between TP53INP1 and TNBC, and we speculate that the existence of VM may create a close linkage among TP53INP1, TNBC and metastasis.

Vasculogenic mimicry plays an important role in cancer progression.^38^, ^39^ VM can provide a blood supply for a malignant tumour, and it also provides a route for tumour cells to escape the primary lesion.^4^ Several molecular mechanisms and signal transduction pathways participate in VM formation.^5^, ^6^, ^40^ In vitro, we found that TP53INP1 knock‐down was associated with the formation of channel‐like structures in breast cancer cells. In addition, the expression of VE‐cadherin, a hallmark of VM, was reduced in TP53INP1‐overexpressing breast cancer cells. The expression levels of MMP2 and MMP9, two important members of the MMP family that participate in extracellular matrix remodelling and VM formation,^41^ were decreased in TP53INP1‐overexpressing BCC. Furthermore, we confirmed that TP53INP1 inhibits VM formation in TA2 mice, which may offer a new therapeutic approach for clinical therapy.

Recent studies have shown that EMT, in which the cell‐cell junction is lost and cells lose the epithelial phenotype and acquire mesenchymal features, is a critical step in the process of cancer metastasis.^42^ Additionally, EMT plays a crucial role in VM formation.^11^ In our study, we tested whether EMT participates in the inhibition of VM formation by TP53INP1. We found that EMT protein marker levels changed in these TP53INP1‐transfected cell lines in vitro and in vivo. Meanwhile, the knock‐down of TP53INP1 resulted in the acquisition of a fibroblast‐like morphology. Thus, TP53INP1 may be a key molecule involved in the breast cancer EMT process.

HIF‐1α is activated by the chemical inducer CoCl_2_, which forces cancer cells to adapt to a hypoxic environment, speeding up VM formation and EMT.^10^ In our CoCl_2_‐induced hypoxia cell model, we confirmed that overexpressing TP53INP1 hindered breast cancer invasion and the formation of VM vessels; hypoxia‐induced breast cancer cells overexpressing TP53INP1 had decreased expression of VM and EMT‐related proteins. We also found that the suppression of HIF‐1α causes a reduction in VE‐cadherin and MMP2 in hypoxia‐induced overexpressing‐TP53INP1 cells. Cells cotransfected with shTP53INP1 and shHIF‐1α also exhibited decreased levels of VE‐cadherin and MMP2 in hypoxic conditions, suggesting that TP53INP1 is needed for hypoxia‐induced VM formation. We speculated that HIF‐1α acts on one gene locus of the TP53INP1 promoter. However, the relationship between TP53INP1 and HIF‐1α must be studied further.

Accordingly, we needed to determine which pathway was mediated by TP53INP1 to inhibit hypoxia‐induced EMT and VM formation. ROS can promote cancer progression and metastasis by regulating signalling pathways and intracellular metabolism in response to hypoxia.^43^, ^44^ EMT is induced through the activation of many transcription factors, such as Snail, MAPK and PI3K.^43^, ^45^, ^46^ We found high ROS levels in TP53INP1‐deficient cell lines. Moreover, the inhibition of ROS generation by NAC inhibited VM channel formation and VM‐related protein expression. Glycogen synthase kinase 3β (GSK3β), a Ser/Thr protein kinase, is an important node; restricting its activation in epithelial cells can limit cancer progression and metastasis.^47^ Research has suggested that oxidative stress activates GSK3β, which is then involved in inducing the activation of Snail in a hypoxic environment, and the phosphorylation of GSK‐3β promotes the stabilization of Snail.^48^ The levels of GSK‐3β and phosphorylated GSK‐3β were measured to evaluate whether GSK‐3β is involved in VM formation and EMT via the ROS‐mediated pathway. We concluded that GSK‐3β likely participates in the process. Therefore, TP53INP1 can be reasonably assumed to suppress hypoxia‐induced breast cancer EMT and VM formation via the GSK‐3β/Snail pathway. However, we did not rule out other pathways; additional detailed studies must be performed.

In conclusion, we identified a novel function of TP53INP1 that is associated with EMT and VM formation in breast cancer. TP53INP1 inhibits hypoxia‐induced EMT and VM formation via the ROS/GSK‐3β/Snail pathway in breast cancer, especially in triple‐negative breast cancer. Therefore, targeting TP53INP1 may be a promising anti‐angiogenesis strategy for treating this disease.

## CONFLICT OF INTEREST

The authors declare no conflict of interest.

## AUTHOR CONTRIBUTION

XLZ conceived the study. YW and DFZ designed the experiments. YW, HZS and DF carried out the experiments and data collection. YW wrote the manuscript and prepared the figures. YHZ, XYD, SQL and FL provided technical support. CSN, ZY and YLL participated in the histological examination of tissue samples. All authors read and approved the final manuscript.
