# Supplementary material for: TP53INP1 inhibits hypoxia‐induced vasculogenic mimicry formation via the ROS/snail signalling axis in breast cancer
Source: J Cell Mol Med. 2018 Apr 14;22(7):3475–88. doi: 10.1111/jcmm.13625 (PMC6010892; doi:10.1111/jcmm.13625)
Supplement: Supplementary file 1 [file JCMM-22-3475-s001.docx]

**SUPPLEMENTARY INFORMATION：**

# TP53INP1 inhibits hypoxia-induced vasculogenic mimicry formation via the ROS/snail signaling axis in breast cancer

Yi Wang^1#^, Huizhi Sun^1#^, Danfang Zhang^1,2,#^, Dan Fan^1^, Yanhui Zhang^3^, Xueyi Dong^1^, Shiqi Liu^1^, Zhao Yang^1^, Chunsheng Ni^2^, Yanlei Li^1^, Fang Liu^1^, Xiulan Zhao^1,2,*^

Supplemental Figure legends


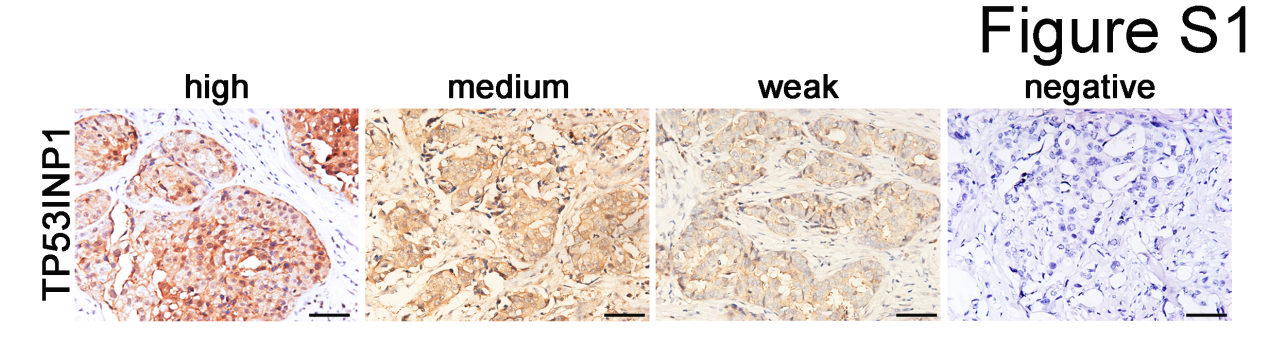


Figure S1. The expression of TP53INP1 in breast cancer tissues. Staining is based on four categories: high, medium, weak, and negative (200×, bar=20 μm).


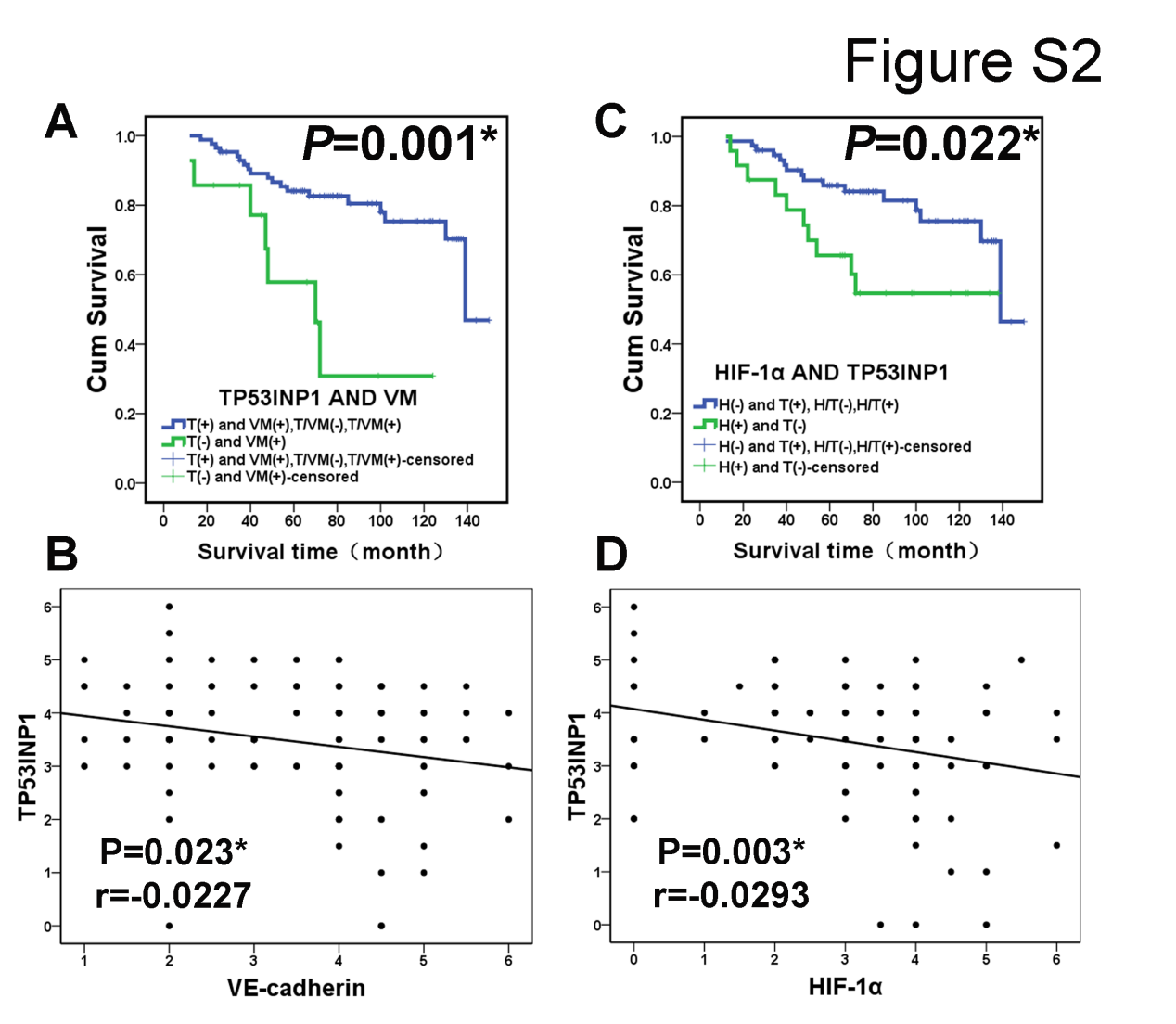


Figure S2. The correlation of TP53INP1 expression with HIF1-α and VE-cadherin. (**A**) A combination of TP53INP1 expression and VM presence was related to poor prognosis by Kaplan-Meier survival analysis (*p*=0.001). (**B, D**) Pearson analysis shows that the expression of TP53INP1 is negatively correlated with the expression of HIF1-α (r=-0.0293, *p*=0.003) and VE-cadherin (r=-0.0227, *p*=0.023) in breast cancer patients. (**C**) Kaplan-Meier survival analysis showed that the combination of TP53INP1 and HIF-1α had poor prognosis (*p*=0.022). **p*<0.05 （T:TP53INP1, H:HIF-1α）


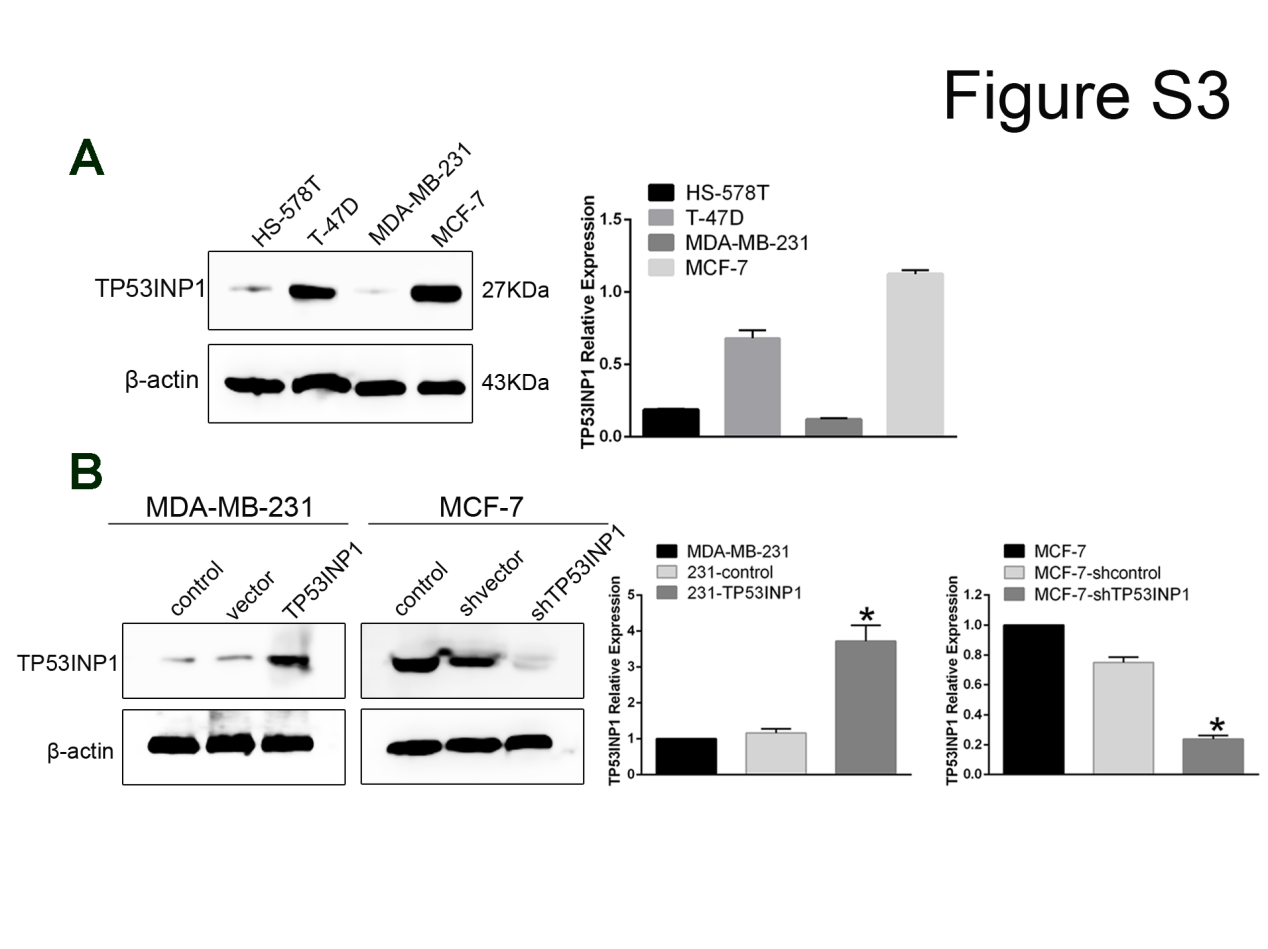


Figure S3. The basal expression of TP53INP1 in breast cancer cells and the establishment of stable TP53INP1 knockdown or overexpression cell lines. (**A**) TP53INP1 protein levels in MDA-MB-231, HS-578T, MCF-7, and T-47D cell lines were evaluated by Western blot analysis. (**B**) The basal expression of TP53INP1 and expression after stable transfection with TP53INP1 cDNA in MDA-MD-231 cells; the basal expression of TP53INP1 and expression in shTP53INP1-transfected and shcontrol-transfected MCF-7 cells. Data are presented as the means ± SD.**p*<0.05


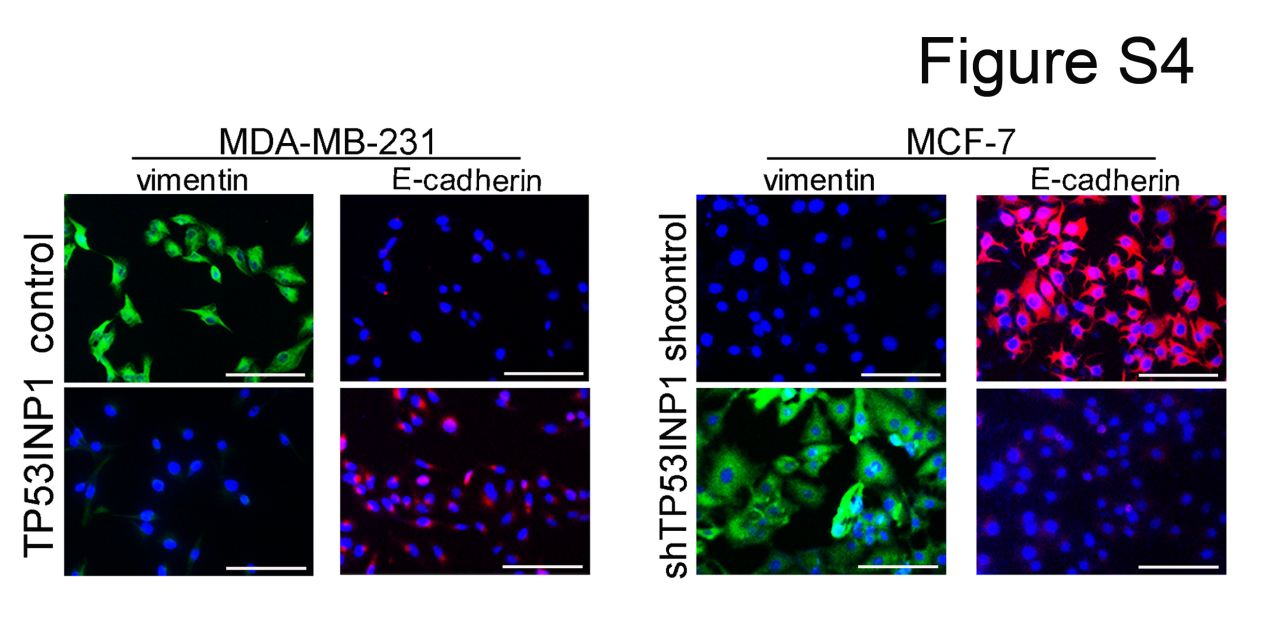


Figure S4. The expression of E-cadherin and vimentin in transfected breast cancer cells by immunoﬂuorescence staining. TP53INP1 overexpression decreased vimentin expression and increased E-cadherin. TP53INP1 silencing had opposite results based on immunoﬂuorescence staining (100×, bar=100 μm).


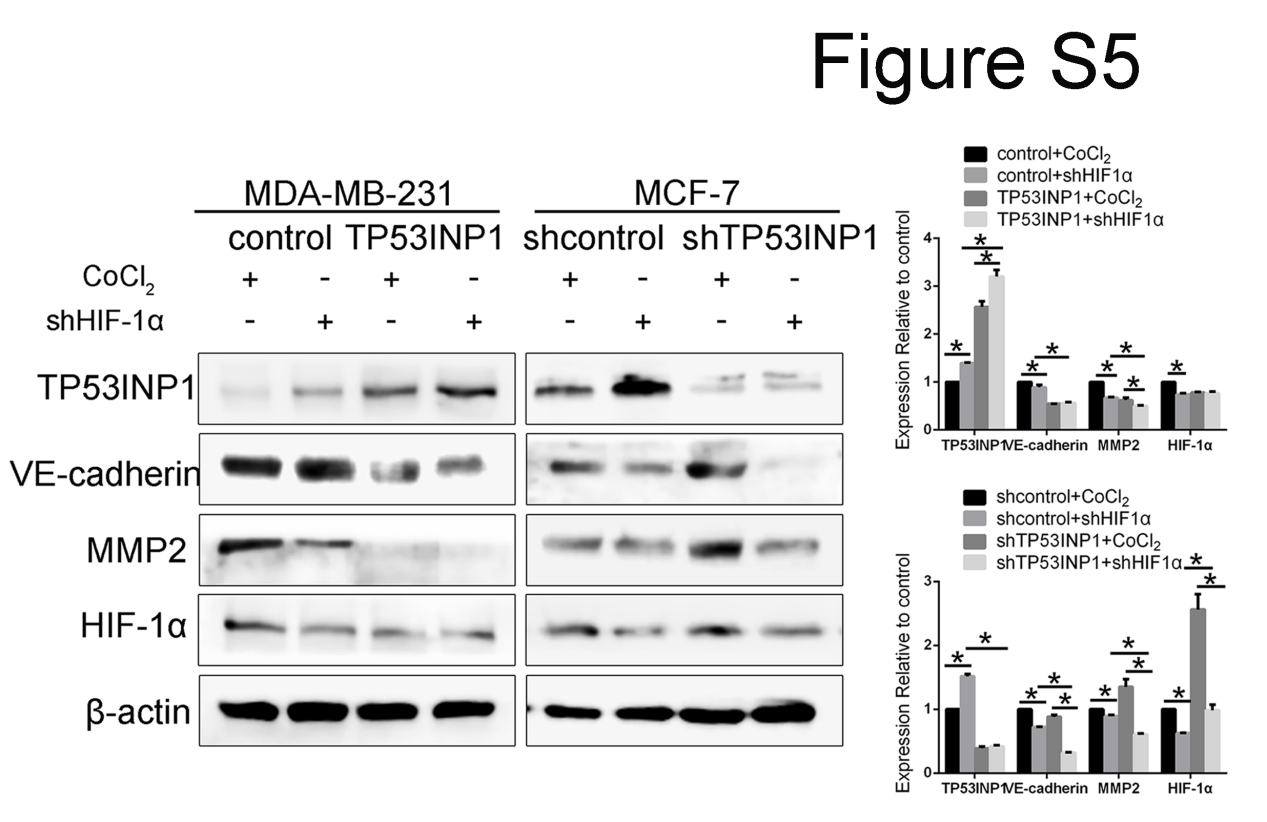


Figure S5. The protein levels of TP53INP1, VE-cadherin, MMP2, and HIF-1α in TP53INP1-overexpressing cells and TP53INP1-silenced cells treated with CoCl_2_ or shHIF-1α. Data are presented as the means ± SD. **p*<0.05

_
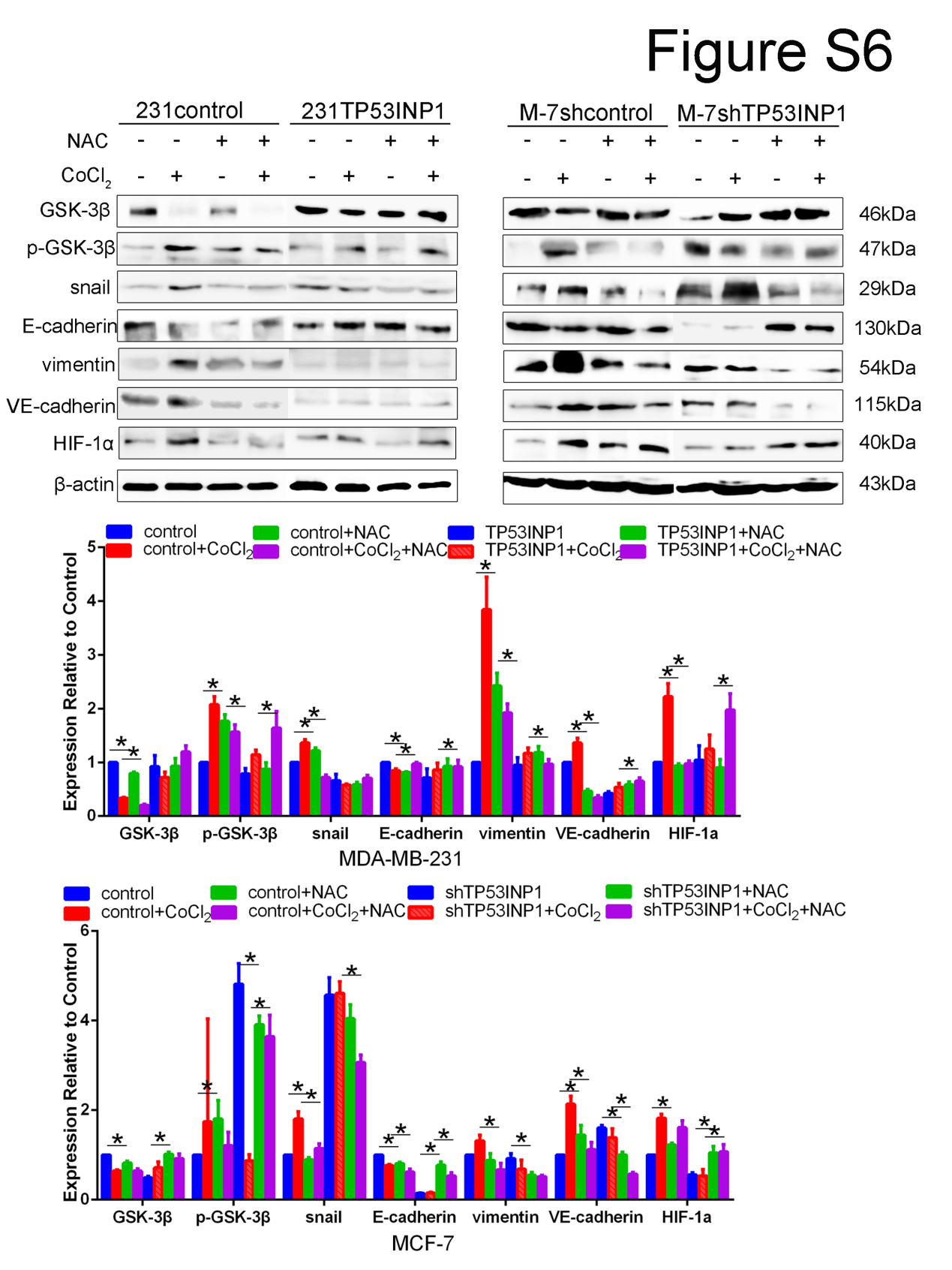
_

Figure S6. The expression levels of GSK-3β, p-GSK-3β, Snail, E-cadherin, vimentin, VE-cadherin, and HIF-1α were tested with NAC or CoCl_2_ treatment in TP53INP1-transfected breast cancer cells by Western blotting assay. Data are presented as the means ± SD. **p*<0.05
