# Supplementary material for: TP53INP1 inhibits hypoxia‐induced vasculogenic mimicry formation via the ROS/snail signalling axis in breast cancer
Source: J Cell Mol Med. 2018 Apr 14;22(7):3475–88. doi: 10.1111/jcmm.13625 (PMC6010892; doi:10.1111/jcmm.13625)
Supplement: Supplementary file 2 [file JCMM-22-3475-s002.doc]

Table S1. Antibodies used in this study

| product name | Manufacture | Concentration | Product number | Source |
| --- | --- | --- | --- | --- |
| TP53INP1 | Abcam | IHC (1:400)  WB (1:2000) | ab202026 | Rabbit |
| snail | Abcam | IHC (1:200)  WB (1:1000) | ab180714 | Rabbit |
| GSK-3β | Abcam | WB (1:5000) | ab32391 | Rabbit |
| *p*-GSK-3β | Abcam | WB (1:3000) | ab75814 | Rabbit |
| E-cadherin | ZSGB-BIO | IHC (1:100)  WB (1:200) | ZS7870 | Rabbit |
| vimentin | Abcam | WB (1:500) | ab92547 | Rabbit |
| vimentin | Santa | IF(1:200) | sc-6260 | Mouse |
| HIF-1a | Abcam | WB (1:10000) | ab92498 | Rabbit |
| HIF-1a | Santa | IHC (1:100) | sc-13515 | Mouse |
| VE-cadherin | Abcam | IHC (1:100)  WB (1:1000) | ab33168 | Rabbit |
| VE-cadherin | Santa | IF(1:200) | sc-52751 | Mouse |
| MMP2 | LuoSai-BIO | IHC (1:100)  WB (1:1000) | 10373-2-AP | Rabbit |
| MMP9 | Abcam | 1:500 | ab76003 | Rabbit |
| β-actin | sc1616-R | 1:2000 | Santa Cruz | Rabbit |

Table S2. The sequences of primer used for RT-PCR

| Gene | Forward primer（5'-3'） | Reverse primer （5'-3'） |
| --- | --- | --- |
| HIF-1α | 5’-GTCGGACAGCCTCACCAAACAGAGC-3’ | 5’-GTTAACTTGATCCAAAGCTCTGAG-3’ |
| CDH5 | 5’-ACAAGGACATAACACCACGAAACG-3’ | 5’-TGAGATGACCACGGGTAGGAAGT-3’ |
| GAPDH | 5’-CCTGGCCAAGGTCATCCATGAC-3’ | 5’-TGTCATACCAGGAAATGAGCTTG-3’ |
